# Challenges and opportunities associated with neglected tropical disease and water, sanitation and hygiene intersectoral integration programs

**DOI:** 10.1186/s12889-015-1838-7

**Published:** 2015-06-11

**Authors:** E. Anna Johnston, Jordan Teague, Jay P. Graham

**Affiliations:** The George Washington University Milken Institute School of Public Health, 950 New Hampshire Ave, NW Suite 400, Washington, DC, 20051 USA; WASH Advocates, 1506 21st Street NW, Suite 200, Washington, DC, 20036 USA

**Keywords:** Neglected tropical disease, NTDs, Water, sanitation, and hygiene, WASH, Program, Integration, Collaboration, Implementation

## Abstract

**Background:**

Recent research has suggested that water, sanitation, and hygiene (WASH) interventions, in addition to mass drug administration (MDA), are necessary for controlling and eliminating many neglected tropical diseases (NTDs).

**Objectives:**

This study investigated the integration of NTD and WASH programming in order to identify barriers to widespread integration and make recommendations about ideal conditions and best practices critical to future integrated programs.

**Methods:**

Twenty-four in-depth, semi-structured interviews were conducted with key stakeholders in the global NTD and WASH sectors to identify barriers and ideal conditions in programmatic integration.

**Results:**

The most frequently mentioned barriers to WASH and NTD integration included: 1) differing programmatic objectives in the two sectors, including different indicators and metrics; 2) a disproportionate focus on mass drug administration; 3) differences in the scale of funding; 4) siloed funding; and 5) a lack of coordination and information sharing between the two sectors. Participants also conveyed that a more holistic approach was needed if future integration efforts are to be scaled-up. The most commonly mentioned requisite conditions included: 1) education and advocacy; 2) development of joint indicators; 3) increased involvement at the ministerial level; 4) integrated strategy development; 5) creating task forces or committed partnerships; and 6) improved donor support.

**Conclusions:**

Public health practitioners planning to integrate NTD and WASH programs can apply these results to create conditions for more effective programs and mitigate barriers to success. Donor agencies should consider funding more integration efforts to further test the proof of principle, and additional support from national and local governments is recommended if integration efforts are to succeed. Intersectoral efforts that include the development of shared indicators and objectives are needed to foster conditions conducive to expanding effective integration programs.

**Electronic supplementary material:**

The online version of this article (doi:10.1186/s12889-015-1838-7) contains supplementary material, which is available to authorized users.

## Background

Recent policy and advocacy efforts have focused on the need to move toward programmatic integration of NTD and WASH activities in order to achieve long-term elimination of NTDs and diarrheal diseases. Research conveys that gains made from Mass Drug Administrations (MDAs) cannot be sustained without some level of investment in water, sanitation, and hygiene. Efforts to identify evidence-based recommendations on how to best integrate NTH and WASH programming in the field have been limited.

### The Burden of NTDs and the Link to WASH

The burden of NTDs across the globe is extensive. In 2013, the World Health Organization (WHO) reported that at least on NTD is endemic in 149 countries, and hundreds of millions of people require treatment [1]. NTDs are also the most common group of infections in the world’s most marginalized people, particularly affecting those known as the “bottom billion” ([2]; Hotez et al. [3]).

The six most common NTDs include Soil-transmitted Helminths (STHs), specifically roundworm (Ascaris lumbricoides), whipworm (Trichuris trichiura) and hookworms (Necator americanus and Ancylostoma duodenale), Schistosomiasis, Trachoma, and Lymphatic Filariasis (LF). These diseases affect one sixth of the world’s population with 90 % of the disease burden occurring in sub-Saharan Africa [4]. Twenty-four percent of the global population is infected with STHs making that group the most common among NTDs [5]. In 2013, 890 million individuals were at risk for STH infection, while only 31 % of people at risk were receiving treatment [6]. As a result STH contributes to over four million disability adjusted life years (DALYs) globally [5]. Furthermore, World Bank research has estimated that as much as 50 % of undernutrition is associated with infection with intestinal parasites or repeated episodes of diarrhea as a result of insufficient WASH [7].

Schistosomiasis is the second most common NTD; 90 % of all these infections affect children, adolescents, and young adults in sub-Saharan Africa. People infected with Schistosomiasis are more likely to be anemic, under-nourished, and stunted, leaving them more vulnerable to other health complications [8]. Women infected with genital Schistosomiasis have been found to have a three-fold increased risk of becoming infected with HIV [9].

Trachoma, the world’s leading cause of preventable blindness, contributes to an estimated 3 to 6 billion U.S. dollars in lost productivity every year ([10] (1); [11] (2)). Trachoma is endemic in 59 countries, but just 14 countries^1^ comprise 80 % of the total burden [12]. Trachoma and Schistosomiasis can both be averted with access to adequate WASH [13, 12, 14]. Use of improved sanitation has been shown to reduce Schistosomiasis and Trachoma by 77 and 27 %, respectively [15].

There are 120 million people currently living with LF across the globe, 40 million of which are already suffering from disfigurement, and over half of them reside in Southeast Asia [1, 16]. LF causes severe swelling and disfigurement that can potentially lead to permanent disability. While the necessary drugs to treat LF are donated by pharmaceutical companies treatment coverage remains low according to the WHO; only three percent of the at-risk population was reached in 2012 [17]. Disability associated with LF can be prevented with improved sanitation and hygiene [18].

Various strategies have been developed by the WHO that aim to hasten the control and elimination of some of the aforementioned diseases. Table 1 shows the NTDs, current global control and elimination strategies and how WASH interventions can affect NTD outcomes. A recently published implementation guide developed by a consortium of NTD and WASH organizations adds to the limited literature that encourages integration of WASH and NTD activities [19]. Tables 2 and 3 display the links and potential impact WASH interventions could have on NTDs. It is likely that a more holistic approach to NTDs and WASH efforts will benefit both sectors along with the communities they are aiming to serve. This is especially true in areas that are endemic with more than one NTD [19].

**Table 1:** World Health Assembly (WHA) resolutions and global programs targeting NTDs

Disease	World Health Assembly (WHA) Resolutions and Global Programs
Soil-transmitted helminths (STH)	WHA 54.19 (2001): Goal of a minimum of 75 % of school-aged children receiving regular chemotherapy by 2010; encouraging member states to promote access to safe water, sanitation and health education through inter-sectoral collaboration.
Schistosomiasis	WHA 54.19 (2001): Goal of a minimum of 75 % of school-aged children receiving regular chemotherapy by 2010; encouraging member states to promote access to safe water, sanitation and health education through inter-sectoral collaboration
	WHA 65.21 (2012): Encouraged member states to provide necessary and sufficient means and resources for water, sanitation, and hygiene interventions in order to achieve elimination.
Trachoma	WHA 51.11: Established goal of eliminating blinding trachoma. Includes call for implementation of facial cleanliness and environmental improvements as part of SAFE strategy
Lymphatic Filariasis	WHA 50.29 (1997): Elimination of LF as a public health problem. Includes a call for increased access to safe water, sanitation, and health education through intersectoral collaboration.
	Global Program to Eliminate LF (GPELF) (2000): Launched to eliminate LF by 2020. Strategy based on interrupting transmission through MDA and alleviating suffering through morbidity management and disability prevention.
Guinea Worm	WHA 64.16: Calls on all Member States to expedite the interruption of transmission and enforce nation-wide surveillance to ensure eradication of Guinea World disease.

Permission was granted from an author of the manual below. Global Policies for the NTDs, 2013. Available from: http://washntds.org/PDF/ALL%20WASH%20NTD%20Manual.pdf Table showing the list of World Health Assembly resolutions that specifically target NTDs

**Table 2:** The link between water, sanitation, and hygiene interventions and NTDs

Type of Intervention	Specific Intervention	Diseases Impacted
Water	Increasing access to sufficient amounts of safe water for personal hygienic purposes (e.g., washing hands, face, or body; bathing; and doing laundry)	Soil-transmitted helminthiasis, Schistosomiasis, Trachoma, Lymphatic Filariasis, Guinea worm disease
	Increasing access to sufficient amounts of safe water for environmental sanitation (e.g., cleaning latrines)	Soil-transmitted helminthiasis, Schistosomiasis, trachoma
	Increasing access to safe water for drinking/food preparation	Guinea Worm disease, soil-transmitted helminths
	Monitoring impact of water resource development, waste water management, and sanitation programs on vector breeding levels	Schistosomiasis, Lymphatic Filariasis
Sanitation	Reducing open defecation	Soil-transmitted helminthiasis, Schistosomiasis, Trachoma
	Disposing of infant/child feces properly	Soil-transmitted helminthiasis, Schistosomiasis, Trachoma
	Increasing improved sanitation coverage	Soil-transmitted helminthiasis, Schistosomiasis, Trachoma
	Promoting maintenance and cleaning of latrines	Soil-transmitted helminthiasis, Schistosomiasis, Trachoma
Type of Intervention	WASH Messaging	Diseases Impacted
Hygiene	Hand washing	Soil-transmitted helminthiasis
	Face washing	Trachoma
	Wearing shoes outside	Soil-transmitted helminthiasis
	Daily washing, with soap, of swollen limbs, feet, and between toes to prevent bacterial infections	Lymphatic Filariasis
	Washing of soiled clothing/bedding	Trachoma
	Avoiding physical contact with contaminated surface water	Schistosomiasis
	Use of safe water for bathing, clothes washing, and swimming	Schistosomiasis
	Avoiding physical contact with or entering bodies of water used for drinking	Guinea Worm disease

Permission was granted from an author of the manual below. Water and Sanitation Interventions, 2013. Available from: http://washntds.org/PDF/ALL%20WASH%20NTD%20Manual.pdf Hygiene Interventions, 2013. Available from: http://washntds.org/PDF/ALL%20WASH%20NTD%20Manual.pdf Table that shows the WASH and other social interventions and their links to NTDs

**Table 3:** Impact of WASH on NTDs

WASH objectives for disease control	Enabling activities	Desired behaviors	NTD-specific outcomes
Reduced amount of human feces in environment	Construction and maintenance of latrines	Elimination of open defecation practices	Reduced breeding sites for the *M. sorbens* fly, which spreads trachoma
			Reduced transmission of STH and schistosome eggs
Daily practice of personal and environmental hygiene activities	Increased access to water in homes, schools and communities	Increased daily hand washing behaviors at key times	Elimination of bacteria and eggs from hands
	Behavior change communication	Increased daily face washing	Reduced reservoir of trachoma bacteria transmitted via flies, fingers, and fomites
		Decreased contact with contaminated surface water bodies	Separation of people from water infested with schistosome parasites
		Increased use of safe water for washing clothes, bathing, and swimming	Separation of people from water infested with schistosome parasites
		More frequent washing of clothes in safe water	Reduced transfer of trachoma bacteria via dirty fabric
		Cleaning and upkeep of latrines	Reduced breeding sites for the *M. sorbens* fly, which spreads trachoma
		Increased washing of lower limbs and feet affected by lymphedema	Removal of dirt and bacteria that can cause skin infections

Permission was granted from an author of the manual below. Impact of WASH on the NTDs, 2013. Available from: http://washntds.org/PDF/ALL%20WASH%20NTD%20Manual.pdf Table shows more specified impact that WASH has on NTD prevention

A major limitation of integration efforts to date is the lack of evidence that links precise integrated approaches to reductions in targeted NTD outcomes. While it is widely accepted that WASH interventions are essential in preventing STH infection and that MDAs alone will not protect people from re-infection as stated, there is limited evidence to determine which specific intervention is most effective and efficient for reducing STH [20, 18]. This gap in evidence affects the willingness of donors, NGOs and governments to invest in integrated programs. The most well recognized NTD control and elimination plan, that integrates WASH, is the SAFE strategy developed for Trachoma. SAFE advocates for surgery, antibiotics, facial hygiene and environmental change to control and eliminate Trachoma [21, 22]. Although SAFE arguably gives Trachoma a leg up in comparison to other NTDs the integrated strategy does not provide specifics in terms of targets or best practice interventions supported by the SAFE framework [23]. There are recent efforts to integrate NTDs and WASH programs, yet very little research has been conducted to assess how integration efforts are progressing, or not, and which factors drive their success or failure. This study investigates the integration of NTD and WASH programming in order to identify barriers to widespread integration and make recommendations about ideal conditions and best practices critical to future integrated programs.

## Methods

Key stakeholders in the WASH and NTD fields were identified based on their work or research in the NTD and WASH sectors. The initial potential interview participants were identified as partners working with the Global Network for Neglected Tropical Disease. They represented both the NTD and WASH sectors in a variety of locations. These individuals were contacted and were either recruited for an interview or asked to recommend other individuals who were more appropriate.

From the initial interviews snowball sampling was utilized to find additional interview participants who worked in organizations that were known to integrate projects or were known to collaborate with other organizations in NTD and WASH programming. Individuals in either headquarters or field positions, with detailed knowledge about their organization’s implementation process, were interviewed. In-depth, semi-structured interviews were conducted using a detailed interview guide, which can be found in Additional file 1: Appendix 1.

For the purposes of this analysis key stakeholders included donor organizations, United Nations agencies, international NGOs, and academic institutions. It does not include feedback from in-country Ministry representatives that are connected to NTDs or WASH or program beneficiaries. National ministry stakeholders were not included in the current analysis.

Twenty-four participants were interviewed beginning in November 2013 through February 2014 by the first author. These interviews were given in person and through video calls. All of the interviews were audio recorded using AudioNote 4.0.3 Luminant Software. The interviews were then transcribed and uploaded to NVivo 10 for Mac version 10.0.3 to be coded and analyzed for themes. The coding was completed using the order of the interview guide along with the interview transcriptions. Barriers were discussed first and key words or phrases were identified in transcriptions by their theme and coded in NVivo. The same was done for ideal conditions. The most commonly mentioned codes were identified after all the coding was completed. These will be highlighted later in the analysis.

The interview participants who were identified as implementing NTD and WASH collaboration or integration programs were also given a two page quantitative questionnaire to fill out after the interview. This questionnaire was used to ensure that the authors had a detailed understanding of all components of the interviewee’s NTD and WASH programming activities. These data were compiled and reviewed using Excel for Mac version 14.4.1. Table 4 contains background information of study participants, by area of expertise, location of work, and position within organization where they were employed.

**Table 4:** Background information on study participants

Area of expertise	N ( %)
NTDs	11 (45 %)
WASH	7 (29 %)
Environmental health	2 (8 %)
School health	2 (8 %)
Behavior change	1 (4 %)
Community health	1 (4 %)
Location	
Headquarters	16 (66 %)
Field	8 (33 %)
Position	
Technical advisor	9 (37 %)
Operations / Managing director	9 (37 %)
Research associate	2 (8 %)
Policy analyst	1 (4 %)
Program associate	1 (4 %)
Program manager	1 (4 %)
WASH/NTD coordinator	1 (4 %)

Table provides more information on study participants, including area of expertise, location, and job titles

### Ethics statement

This study was approved by The George Washington University Human Subjects Internal Review Board (IRB #101309). All of the interview participants in this study were adults. All interviewees were provided with an informed consent form via email prior to their interview that was approved by The George Washington University Human Subjects Internal Review Board. Their consent was given originally in a response email and then was reiterated orally before any interview questions were asked.

## Results

All 24 interviews explored possible barriers or challenges to intersectoral collaboration of NTD and WASH programs. Participants also identified and discussed several needs or ideal conditions for future integration efforts. Both the barriers and the ideal conditions discussed in this section were the most commonly stated themes among interview participants, regardless of sector of work or type of organization. The principal barriers and ideal conditions are characterized below.

### Barriers

The interview participants revealed several important insights regarding the barriers that their organizations face when trying to implement NTD and WASH integration programs. While existing evidence supports the integration of NTD and WASH programing in efforts to reach disease control and elimination, and thereby improving the health of communities, there are several challenges that were consistently reported to make intersectoral collaboration difficult. The most frequently mentioned barriers include issues with differing programmatic objectives, indicators and metrics, an over emphasis on MDA, discrepancies in funding and siloed funding, and lack of coordination and information sharing between sectors. Table 5 summarizes the top barriers to integration cited by participants.

**Table 5:** Barriers to integration identified by study participants

Barriers	N ( %)
Different Programmatic Objectives	17 (71 %)
Indicators and Metrics	14 (58 %)
Over Emphasis on MDAs	12 (50 %)
Funding Discrepancies	11 (46 %)
Coordination & Information Sharing, Lack of	10 (42 %)
Siloed Funding	10 (42 %)
Evidence Base, Lack of	9 (38 %)
Timeline Discrepancies	9 (38 %)
Behavior Change	8 (33 %)
Joint Mapping, Lack of	7 (29 %)
Ministerial Coordination, Lack of	7 (29 %)
Political Will, Lack of	7 (29 %)
Ill Committed Partnerships	5 (20 %)
Government Ownership, Lack of	4 (17 %)
Difference in Results Timelines Between Sectors	3 (13 %)
Joint Messaging, Lack of	3 (13 %)

Table provides the full list of barriers identified by interview participants

#### Differing programmatic objectives

Differing programmatic objectives was the barrier most commonly mentioned by interview participants. Seventeen of the 24 interview participants (71 %) specifically referenced the varied objectives between sectors as a barrier to their work. This particular barrier was insightful in that most participants referenced organizational objectives while some mentioned donor objectives. Illustrative of this point, one key informant stated:

*Participant 23: That is a challenge, the WASH organizations have their own objectives, they have their own donor goals.*

A primary issue that was highlighted was the need for the NTD and WASH sectors to have a better understanding of the others’ priorities, highlighted by this point:

*Participant 16: It’s clear how WASH is important to the NTD community, but the issue is making NTDs a priority to the WASH community.*

Several interviewees, as demonstrated in the quote below, mentioned that some individuals working in the WASH sector are focused on engineering and supplying water and sanitation hardware, and are therefore unaware of the link their work has to the health sector, particularly to NTDs.

*Participant 24: They are sectors that come from very different backgrounds, water and sanitation being very much based in engineering and ourselves being based in more of a biology-medicine area. I think that actually working across sectors and really understanding the motivations of different programs is quite challenging. And I think we perhaps underestimate the kind of work you have to do to create meaningful relationships and collaborations.*

#### Indicators and metrics

Issues around indicators and metrics were listed as a barrier by 14 of the interview participants (58 %). Participants explained that different monitoring and evaluation measurements sometimes made it impossible for organizations to integrate their NTD and WASH programming. This challenge links back to the first barrier in that priorities and objectives manifest themselves in desired impacts and indicators of success.

*Participant 12: WASH organizations usually don’t measure health indicators.*

*Participant 7: WASH interventions are the most sustainable way to prevent NTDs. It’s the work they are already doing; they are just not evaluating the impact of that work on NTD outcomes, which is a great lost opportunity.*

While several participants noted that developing joint indicators for both sectors to use is a challenging task most agreed that both sectors stood to benefit from joint indicators over time.

*Participant 9: I agree that metrics are probably the biggest hang-up and probably the most difficult conversation to have, not because it’s sort of a question of intractable differences between the two sides in that respect, but more just what would those shared metrics be and how would you find a common metric between them?*

*Participant 10: It’s really hard to come up with an integrated indicator.*

*Participant 13: The NTD community needs to do a better job of defining those indicators and also making them practical for other sectors to access and understand.*

#### Over-emphasis on mass drug administration

The over emphasis on MDA in NTD control and elimination efforts is an important barrier that was mentioned by half of the 24 interview participants. This barrier can also be linked back to issues related to varied programmatic objectives between the sectors, where WASH implementers tend to focus on preventative services and those working in NTDs tend to focus on curative services.

*Participant 3: I think the big challenge within the whole NTD sector is that until fairly recently all you would hear about in these meetings is preventive chemotherapy. Again it sort of goes in the sense that a lot of public health is overseen by medically trained people and the idea of giving medication is right up their alley, they understand it better, it's reinforced by donors like USAID, and its much easier to say that X number of tablets were distributed than looking at some of the more difficult aspects to define, like behavior change.*

*Participant 19: We need to make sure that WASH is part of the overall plan for NTD elimination in a country, and we need to move away from thinking of NTD programs just as Mass Drug Administration programs.*

Several interviewees pointed out that integration programs should have preventive services paired equally with curative services, and should not be viewed as delivering multiple drugs to locations that are endemic with more than one NTD.

*Participant 15: Integration needs to be more than just collaborating MDAs with multiple drugs.*

*Participant 3: I think in a nutshell one of the major problems we’ve had is that we’ve been taking a very medical approach to very much a public health problem… and not looking at those elements that are really going to sustain the progress we’ve made through the preventive chemotherapy.*

#### Funding

Funding was identified as a barrier in two ways. One in funding discrepancies, which were described as issues around WASH programming being very costly compared to NTD programming; and two in siloed funding, which was described as funding restrictions typically imposed by donors that prevent integrated programming from occurring without a separate funding stream. Eleven and ten interview participants (46 and 42 %) identified challenges associated with funding discrepancies and siloed funding, respectively.

*Participant 23: WASH is very, very resource intensive.*

*Participant 21: Disparities in budget in WASH projects versus NTD projects make it difficult for both sectors to work together in a specific location.*

Siloed funding hinders NTD and WASH programmatic integration because it greatly restricts the type of programming that an organization can implement without additional funding streams. Both sectors identified issues associated with siloed funding equally.

*Participant 11: We get money for Neglected Tropical Diseases, we don’t get money to build toilets. That’s not our mandate. So we coordinate with people that build toilets, but that’s not the same as having our own money to build toilets, which means we can’t really have comprehensive programs on the ground.*

Even organizations that are typically viewed as donors struggle with siloed funding.

*Participant 1: You see because the thing is that’s why this is an interesting topic, this whole WASH and NTD integration because its not easy, and one of the problems that we have is, you know, very specific funds. We get funds from Congress to do very specific things.*

Participants were not only concerned about the amount of funding, but also the mechanism of funding and how limiting it is on program implementation. Even if there are various funding streams that allow for implementation of an integrated program, which is relatively rare, it is still a challenge to coordinate those streams into one program design without considerable synchronization.

#### Lack of coordination and information sharing

Ten of the interview participants (42 %) identified lack of coordination and information sharing as a barrier to NTD and WASH integration. Interviewees explained that efforts to share information and coordinate programming accordingly would make integrating projects more successful. This type of coordination and information sharing is a challenge for large organizations with a broad spectrum of programs. According to participants, information often is not shared because there is no specific mandate to do so. Employees at these organizations are often over stretched and do not independently prioritize taking time to share information with other organizations.

*Participant 6: We’re not doing any mapping or targeting between those two initiatives. With our pharma procurement we’re not looking at the countries where we have significant WASH programming and trying to build that investment on top of that programming, which is again a huge lost opportunity.*

*Participant 7: We are procuring a tremendous quantity of deworming medication…and distributing that with our Ministry of Health partners, but as an investment it is not being purposely leveraged together with our WASH programming.*

It is also a challenge for more specialized organizations that try to work together to implement integrated programs.

*Participant 21: In terms of countries and areas [within countries] that are highly endemic, we don’t necessarily always match up with areas that have been targeted for prioritization of infrastructure and water and sanitation projects by other organizations, which is a huge barrier.*

Interview participants that discussed this barrier often mentioned that coordination and information sharing should occur between multiple parties to ensure that the benefits of integration can be exploited, and that parties involved in funding mechanisms should encourage integration.

*Participant 15: So something needs to change higher up and when I say higher up it includes the big aid agencies, it includes the pharma companies, it includes foundations, it includes everybody.*

### Ideal conditions

During their interviews participants discussed several ideal conditions that they believe would allow for NTD and WASH programmatic integration to occur more freely. The most frequently mentioned ideal conditions include educational advocacy, development of joint indicators, increased ministerial involvement, integrated strategy development, creating Task Forces or committed partnerships, and improved donor environment for funding integration. Table 6 contains a list of all 12 ideal conditions sited by interview participants. This section will highlight the six barriers that were identified and discussed most frequently by interview participants. Four ideal conditions highlighted in this analysis were identified by 12 of the interview participants, so they were also ranked by the number of times they were mentioned in transcribed interviews in order to position them appropriately.

**Table 6:** Ideal Conditions to integration identified by study participants

Ideal conditions	N ( %)
Educational Advocacy	17 (71 %)
Joint Indicators	13 (54 %)
Ministerial Involvement	12 (50 %)
Integrated Strategy Development	12 (50 %)
Task Force or Committed Partnership	12 (50 %)
Donor Environment for Funding Integration	12 (50 %)
Appropriate Programmatic & Results Timelines	11 (46 %)
Government Ownership	11 (46 %)
Information Sharing & Message Integration	11 (46 %)
Program Design Integration	11 (46 %)
School Curriculum Integration	11 (46 %)
Evidence-based Best Practices	10 (42 %)
Joint Mapping	9 (38 %)
Funding Advocacy	8 (33 %)
Geographic Overlap Targeting	8 (33 %)
Linking NTDs & WASH to Nutrition	7 (29 %)

Table provides the full list of ideal conditions identified by interview participants

#### Educational advocacy

Educational advocacy was described as increased knowledge between the NTD and WASH sectors, and was highlighted as an ideal condition for successful integration by 17 key informants (71 %). The interviews suggested that increased knowledge in both sectors, but particularly the WASH sector, could lead to a better understanding of the roles both parties can play in provision of health services.

*Participant 10: WASH, especially sanitation, and NTD sectors are both neglected issues that usually include the same demographic and same population so there is a lot of incentive for both sectors to come together.*

*Participant 13: I think that honing in on the fact that we’re all working to improve the health and lives of the same communities, essentially…so it’s improving those communication channels, awareness levels and knowledge and information sharing across the sectors. I think we’re seeing success with this and we should continue to build on that.*

It was also suggested that this mutual understanding will help standardize the process of designing integrated programs. It could also be an important component in creating guidelines or manuals that could assist in narrowing down integration activities associated with different NTDs.

*Participant 7: Really I think for us to be most successful with this integration our WASH implementers and people involved in the design of our WASH programs need a greater understanding of the NTD issues and NTD indicators need to be standardized in our WASH log frames.*

It was suggested that educational advocacy should work to motivate both sectors to invest in programs that would create mutual success between NTDs and WASH, while also focusing on having the greatest positive impact on the communities where implementation is occurring. Interview participants working in the NTD sector particularly expressed the need to utilize the WASH community in a way that is beneficial and supports both sectors’ desired health outcomes.

*Participant 22: We need to engage them. We need to make them feel that what they are contributing is really valuable.*

#### Joint indicators

Joint indicators were discussed as an ideal condition for successful programmatic integration by 13 interview participants (54 %). The issue of indicators was highlighted twice: the lack of joint indicators being described as a barrier, and dual or exchangeable indicators being described as a condition for successful programs.

*Participant 19: I don’t think we’ve gotten our messaging right. We’ve never given a convincing case to the WASH sector about why they should be looking at these diseases. I’m not a WASH expert, but I believe that they are really looking at things like access to water, improved hygiene, and the general impact on health rather than looking at indicators specific to disease, and I think that one of the reasons why the trachoma and broader NTD communities have found it difficult to work with them.*

*Participant 24: For the water sector their measure of impact have been primarily focused on coverage rather than utilization, and also very few use health markers and if they do they often focus on diarrheal disease rather than NTDs so I think we have some sort of advocacy work there to do to try to convey that actually measuring NTDs as an output or indicator of effective sanitation is very useful.*

The use of joint indicators could be accomplished in several ways. First it could be that both sectors utilize the other’s indicators in the monitoring and evaluation process.

*Participant 5: NTD and WASH programs need to be designed and evaluated simultaneously. So you can call it a WASH program, but NTDs need to be integrated at each step of the way.*

Another option is to use NTD indicators as interim indicators and WASH indicators after some time in the evaluation process. This would be predominantly due to the fact that deworming activities produce results more quickly than WASH activities.

*Participant 7: NTD indicators need to be standardized into WASH log frames, and WASH issues should be standardized into NTD programming.*

The last option is for both sectors to work together to come up with joint indicators. These indicators would ideally be built into the design of a program, and would coalesce the needs of both sectors.

*Participant 13: We’re looking to the WASH community as well to help us to identify what would be the most useful indicators that both sectors could use, and that we’re not collecting data that is only useful to one side or the other. I think it’s important for us to hone in on what those joint indicators would look like.*

Three interview participants who were already implementing programs with joint indicators, highlighted that measurement tools can be representative of the needs of both sectors.

#### Ministerial involvement

The need for ministerial involvement was emphasized as an ideal condition for integration programs. Twelve interview participants (50 %) identified ministerial involvement, and it was cited in transcribed interviews 40 times. Ministerial involvement pertains to the different ministries that potentially need to be included in NTD and WASH programmatic integration at the country level.

*Participant 17: Ministers on the ground really need to facilitate with each other better.*

The Ministry of Health and Ministry of Water are the most obvious of those that should work together in NTD and WASH integration programs. Some countries, however, do not have a Ministry of Water, and in that case it is necessary for the Ministry of Health to see issues around water and sanitation as a part of the public health sphere. If there is a Ministry of Water then it is crucial for those ministries to work together in a very integrated way in terms of projects and geographic locations. It would also be extremely useful for employees of both ministries to have counterparts in the other in order to bring programming together more easily.

*Participant 11: Sometimes it’s kind of like none the two shall meet, they don’t even know where the other person’s office is so then you’re kind of starting from zero.*

*Participant 11: In some of our countries, the Ministry of Health, the Ministry of Education and the WASH department are totally different, but they know each other and they work together on a regular basis. Those are the places where putting a little more effort would have the biggest impact because they already are coordinating. Those are the places that have the biggest potential for integration and success.*

Study participants indicated that other ministries could also need to be included, namely the Ministry of Education. Several interview participants mentioned exploring or implementing integration programs that are school-based. These programs could be short-term lessons or long term messages that are built into the curriculum.

*Participant 10: School based programs that include hygiene education, pill distribution and behavior change efforts are really ideal, but this brings up the issue of being able to coordinate with the Ministry of Education along with the Ministries of Health and Water.*

#### Integrated strategy development

Developing a strategy that includes a deworming and WASH component for the control and elimination of NTDs, similar to the SAFE strategy for Trachoma, was seen as a need by interview participants. Twelve interviewees (50 %) identified integrated strategy development as a requisite, and it was cited in transcribed interviews 34 times. The SAFE strategy, which was developed and is supported by the WHO states that water and sanitation efforts are crucial to Trachoma control and elimination through facial cleanliness and environmental change. Those in the NTD sector more generally explained that a specific strategy, similar to SAFE, should be articulated for the other WASH affected NTDs.

*Participant 15: If we’re talking about integration and the need for it to happen across the board for NTDs then having something like the SAFE strategy for other NTDs would be a good starting point even though it won’t solve everything it at least it brings people together.*

Participants that explored the need to develop an integrated strategy for NTDs affected by water and sanitation believe that it would set up the framework for integration to work more holistically and effectively. Many of them also believed that it would give NTDs like STH, Schistosomiasis and LF control and elimination efforts more momentum. However, several interview participants also recognized that any integrated strategy, including the SAFE strategy, could benefit substantially from operational research aimed at specifying which WASH interventions are best suited to be paired with each NTD in order to maximize integration.

*Participant 13: There needs to be specific articulation of how and why NTD prevention should include WASH; this is why they trachoma community has the upper hand compared to the STH community.*

#### Task force or committed partnerships

Task Force or committed partnership development was discussed as an ideal condition for programmatic integration by 12 interview participants (50 %); it was also stated in interviews 30 times. These partnerships, that were also sometimes referred to as Steering Committees were thought to include different NTD and WASH organizations, relevant ministries in-country, and potentially donors and pharmaceutical companies. Many interview participants explained that this partnership should go beyond a memorandum of understanding; it should institutionalize the way every entity involved works together throughout the duration of a project. This should include a division of roles and responsibilities ideally with the Ministry of Health as the governing body, agreed upon budgets, timelines, and indicators. This process would open the lines of communication, encourage information sharing, and create a situation where each partner is invested in the success of various sectoral objectives.

*Participant 10: The people that were working together to run those programs know each other very well and they are working in the same communities, so its very easy [for them] to see their shared objectives.*

Another interviewee that was involved in a partnership explained that when partners did not follow through with their commitments, either in funding or the project timeline, the partnership and overall programmatic objectives suffered.

*Participant 2: Time and financial commitments [between partners] should be very equal.*

In a situation where NTD and WASH organizations are coming together to create a Task Force to address common issues, appropriate programmatic and results timelines should be established. This key factor should be agreed upon by partners prior to implementation to ensure each side feels invested in mutual success while also maximizing their potential for collaborative impact in an appropriate amount of time for both sectors.

#### Donor environment for funding integration

An improved donor environment, particularly one that allows for funding integration was highlighted as an ideal condition to future integration of NTD and WASH programs. Twelve interview participants (50 %) discussed this need, and it was identified in the transcribed interviews 23 times.

This ideal condition predominantly refers to donor restrictions on integration programs and siloed funding streams that prevent NTD and WASH integration from occurring without another funding source.

*Participant 14: Even if integration is the ideal in order to reach that ultimate goal and common vision of ‘disease free communities’*^2^*it is from a practical standpoint not even possible until there’s a larger commitment among donors.*

*Participant 14: A lot of it really goes back to donors and governments driving that integration in the first place, driving both goals at once.*

Interviewees suggested that for this ideal condition to occur, along with donor commitment a commitment from the national government needs to be achieved. Many interview participants stated that if national governments would begin to prioritize water, sanitation, and NTDs, so would donors. In this way it is essential that governments of countries endemic with one or more NTDs develop a national plan for control and elimination; and equally as important, this plan should include an explanation of the role water, sanitation and hygiene promotion will play in the control and elimination goals. It is fundamental that both sectors be equally prioritized by the national government.

*Participant 22: In most countries these neglected tropical diseases are diseases of marginalized people are not on the highest agenda of the government. Even though they are really, really ugly diseases that cause people to lose their dignity they are not really killing people like malaria and HIV and TB so government tends to put more emphasis on those.*

## Discussion

Much has been published about the impact that WASH interventions can have on the burden of NTDs, and momentum^3^^,^^4^^,^^5^ for the the two sectors to work together has grown significantly over recent months and years. This momentum has come from several large government donors, including the United States and the United Kingdom, along with several NGOs and foundations that work in both the NTD and WASH sectors ([24, 6]; [25]). However, this push for integration has led to a relatively small number of projects being integrated in the field. Common barriers are differing programmatic objectives, over emphasis on MDA, funding discrepancies and siloed funding, and a lack of information sharing. Collectively agreed-upon ideal conditions include the development of shared goals through educational advocacy, government and ministerial inclusion, integrated strategy development, development of committed partnerships, and an improved donor environment for integrated funding. Joint indicators were highlighted as a barrier and a need with a lack of joint indicators and metrics as a significant barrier and developing applicable indicators for both sectors as a need.

New research that focuses on challenges and opportunities associated with integrated global health programing and more specifically WASH and NTD integration and collaboration is supported by the findings of this analysis [26, 27]. Freeman et al. discuss integration of NTD and WASH initiatives and describe the importance of implementers having a common goal and understanding the impact integration can have on health outcomes Ogden et al. [19] also conveyed the view that a “shared vision” established through partnerships, joint advocacy, and fundraising is essential (2013). Major strides can be made in ensuring that both sectors can realize the benefits of integration and collaboration through information sharing and joint mapping. Joint mapping and geographic targeting will identify high-risk areas and a strong evidence base approach that is appropriate for both sectors ([25]; [28]). It will also foster an environment and information base that will allow for the creation of and advocacy tools and partnerships. All of these efforts will help to alleviate the challenge associate with differing programmatic objectives, which was noted by many of the interview participants.

At least a basic level of coordination and information sharing should occur between stakeholders and within organizations. If a deworming program is being implemented in areas where there are poor WASH conditions, hygiene promotion initiatives could be used to maximize the impact of the MDA. Joint mapping and focusing on geographic overlap between organizations and programs should be a tool used in the design stages of a project.

Metrics and measurements as a challenge and the need to develop joint indicators have also been identified in research [24]. Integrating indicators between sectors would be a monumental step toward fully integrating program design, and moreover would promote cohesiveness between sectors. For example, knowledge, attitude and practice surveys could be used to evaluate changes in awareness around hygiene and sanitation; biomedical tests could be employed to measure disease prevalence; coverage surveys could convey the coverage rates of a newly installed improved water source or a school health program that does deworming, but also emphasizes water, sanitation and/or hygiene messages that are applicable to NTDs endemic to the area.

Furthermore, if indicators could be shared between the NTD and WASH sectors, and ideally the education sector as well, projects that aim to update school curricula to include NTD and WASH messaging could more easily be implemented [29]. This is significant because studies have found that health and hygiene education in schools decreases the prevalence and intensity of worm infections in school-going children significantly ([30]; [31]).

The over emphasis on MDA programs has also been cited previously [32]. The prioritization of MDAs as a solution to the NTD problem is understandable, particularly when pharmaceutical companies donate deworming medications. However, while MDAs are a viable and cost effective intervention for NTD control acting as if a medical intervention is the only necessary solution to a public health problem is myopic. It is important to note there are drawbacks to over emphasizing a purely MDA method. When looking at STH specifically, shortcomings include preset coverage targets that are unlikely to achieve the morbidity reduction goal. Moreover, STH is not limited to school-age children and often greatly affects women of childbearing age who are overlooked in this measurement of coverage, and finally resurgence of STH is inevitable without simultaneous improvements in water and sanitation [20, 33, 32]. This research is further supported by the fact that according to the London Declaration, a global commitment to collaborative disease eradication, the international community should focus on “ensuring access to clean water and basic sanitation” and “health education” in NTD control and elimination efforts further supporting that while necessary and important, MDAs alone will not solve the NTD problem. [34].

The importance of ministerial and government involvement has also been cited [29, 19]; [6]. Involving all relevant ministries in the national government is an important step in ensuring that project objectives are met. Involving the host government and ministries also builds capacity and promotes sustainability of the desired outcomes [29]. This type of an effort requires that ministries work outside of their siloes and view some public health needs as a joint responsibility.

The need for donor policy to improve programmatic integration of NTD and WASH programming is recommended. This will help in alleviating some of the burden associated with funding discrepancies and siloed funding. Challenges associated with siloed funding and differing funding amounts between sectors was highlighted as a significant barrier to integration and as a requisite for future progress. As a result there are very few successfully integrated or collaborative projects between the NTD and WASH sectors. Once the donor environment begins to change, organizations and national governments can begin to prioritize integration with appropriate funding, timelines and measurement tools.

The most effective way to support this shift is through operational research that will highlight priority countries through a needs assessment and joint mapping. This will bring more clarity around which specific WASH interventions are best suited to control and prevent each WASH-impacted NTD. This research should initially focus on STH, Schistosomiasis, and Trachoma, as they are the most common and are very clearly linked to WASH. It should also be noted that the Department for International Development (DFID) in the UK has begun funding programs that will integrate NTD and WASH in sub-Saharan Africa, which is a critical step in the right direction. Other donors should also prioritize the impact that MDAs integrated with water, sanitation and hygiene interventions will have on those living in the poorest and most hard-to-reach communities.

### Limitations and strengths

There were certain limitations to this analysis. Primary data were gathered through semi-structured in-depth interviews; therefore, the results are vulnerable to recall bias if certain activities occurred in the distant past. The final sample of interview participants contained slightly fewer WASH practitioners than NTD practitioners, and also contained more headquarter staff than field staff. All interview participants, however, had knowledge of NTD and/or WASH project implementation activities that had occurred or were occurring, and several of the interviewees spent significant time conducting site visits to their respective programs. The authors recognize that the perspective of in-country government officials are critical to integration efforts and future studies should include a diverse set of ministry stakeholders to better understand their perspectives.

This study had several strengths. It validates recently published research around types of barriers and ideal conditions for NTD and WASH integration programs. While there have been very few articles published which contained a general consensus of NTD and WASH stakeholders regarding challenges and needs in integration, this is the first study to the authors’ knowledge where practitioners spoke individually in an anonymous setting allowing them to speak candidly on the subject. The study participants also provided perspectives from a variety of backgrounds, experiences, and locations, and the projects discussed took place in varied geographic locations and utilized various forms of implementation practices.

## Conclusions

While the frequency of NTD and WASH integration programs appears to be increasing, there remain significant efforts to be made. Governments, donors, NTD and WASH implementers can use the feedback given by practitioners to design more informed and better-integrated programs that have the potential to benefit both sectors and their targeted populations. They can also use the data presented here to understand the challenges and needs for effective integration and prepare for potential issues that may arise. Understanding commonly referenced barriers and ideal conditions is an important step toward more effective programmatic integration. Relevant research on programmatic integration completed by Teague et al. [26] found similar results.

Further operational research is needed to determine which individual WASH interventions are best suited for implementation for the specific WASH-related NTDs. It is also recommended that both governments and donors prioritize funding for integrated programs and foster an enabling environment for integration. But ideally these two things should be happening simultaneously. Determining what specific techniques will work best for both sectors through partnerships and the development of shared indicators, alongside operational research should produce applicable and timely results.

## Additional file

Additional file 1:
**Appendix 1: Interview Guide.**

## Abbreviations

NTDs: Neglected Tropical Diseases
WASH: Water, sanitation, and Hygiene
MDA: Mass Drug Administration
WHO: World Health Organization
STH: Soil-transmitted Helminths
LF: Lymphatic Filariasis
DALY: Disability Adjusted Life Year

## Footnotes

1 Burkina Faso, Ethiopia, Sudan, South Sudan, Guinea, Kenya, Mozambique, Niger, Nigeria, Pakistan, Senegal, Tanzania, Uganda, Zambia

2 Stakeholders from the NTD, child health, and WASH sectors convened in 2012 to discuss intersectoral collaboration. Attendees agreed on a common vision that was described as “Disease-free communities that have adequate and equitable access to water and sanitation, and that practice good hygiene.”

3 Uniting to combat NTDs. *London Declaration*. doi: http://unitingtocombatntds.org/resource/london-declaration.

4 WHO, 2012. *Accelerating work to overcome the global impact of neglected tropical disease: a roadmap for implementation.* Geneva, Switzerland: World Health Organization.

5 ANONYMOUS, 2012. *Progress in sanitation needed for neglected tropical diseases*. Lancet 379: 978. doi: 10.1016/s0140-6736(12)60412-7.
