# Supplementary material for: Challenges and opportunities associated with neglected tropical disease and water, sanitation and hygiene intersectoral integration programs
Source: BMC Public Health. 2015 Jun 11;15:547. doi: 10.1186/s12889-015-1838-7 (PMC4464235; doi:10.1186/s12889-015-1838-7)
Supplement: Additional file 1: — Appendix 1: Interview Guide. [file 12889_2015_1838_MOESM1_ESM.doc]

**Appendix 1: Interview Guide**

Participant Information

| Name of the organization |  |
| --- | --- |
| Job title |  |
| Role in programs |  |
| Expertise |  |
| What is the focus of your organization? |  |
| In which countries is your organization active? |  |
| What is the target population of your programs? |  |

**Interview Guide:**

Integration Activities and Successes

1. What NTD and WASH activities have been integrated at this organization?
   1. Specifics: Market-based approaches? Processes? Technologies?
   2. How are the activities integrated?
   3. Do you use partner organizations?
      1. If you do use partner organization(s) for NTD and WASH programs what is the role they play? Is it an essential role?
2. How does your organization define ‘success’ in the NTD/WASH programs?
   1. How is it measured?
3. What integration activities have been successful?
   1. Why were they successful?
   2. Were they repeated elsewhere?
4. How are these activities decided upon?
   1. What processes in headquarters or field offices?

Challenges in Integration

1. What barriers or challenges have your program(s) encountered in integration?
   1. What was the nature of the barrier(s)? Were they localized in the community setting or were they policy or funding related?
   2. Why and how did these affect integration?
2. What did your organization do to overcome those barriers or challenges?
   1. Has your organization changed its processes, activities, etc., in order to prevent the barriers or challenges?
   2. What changes were made?
   3. Has your organization seen success after this change was made?
3. How are these activities decided upon?
   1. What processes in headquarters or field offices?
4. Integration is complex; what are some examples of unforeseen challenges in integrating the programs and solutions for overcoming them?
   1. Example: Households are overwhelmed with too many behaviors just from handwashing, and with integration we add even more new behaviors for them to do – how do your programs balance integration activities with the understanding that it is overwhelming?

Ideal Integration of NTDs and WASH

1. What would ideal conditions be in order to integrate NTD and WASH activities and programs?
   1. Funding
   2. Behavior change – community level
   3. Time
   4. Policies
   5. Process – community level
2. How could or should integration of NTD prevention and WASH look under these ideal conditions?
3. What are your recommendations for how to integrate NTD and WASH activities, based on your experiences?

Thank you for your participation. I will send you a copy of the final report when it has been completed.
